# Protection Enhances Community and Habitat Stability: Evidence from a Mediterranean Marine Protected Area

**DOI:** 10.1371/journal.pone.0081838

**Published:** 2013-12-11

**Authors:** Simonetta Fraschetti, Giuseppe Guarnieri, Stanislao Bevilacqua, Antonio Terlizzi, Ferdinando Boero

**Affiliations:** 1 Department of Biological and Environmental Sciences and Technologies, University of Salento, Consorzio Nazionale Interuniversitario per le Scienze del Mare, Lecce, Italy; 2 Institute of Marine Sciences of National Research Council, Genova, Italy; Université du Québec à Rimouski, Canada

## Abstract

Rare evidences support that Marine Protected Areas (MPAs) enhance the stability of marine habitats and assemblages. Based on nine years of observation (2001–2009) inside and outside a well managed MPA, we assessed the potential of conservation and management actions to modify patterns of spatial and/or temporal variability of *Posidonia oceanica* meadows, the lower midlittoral and the shallow infralittoral rock assemblages. Significant differences in both temporal variations and spatial patterns were observed between protected and unprotected locations. A lower temporal variability in the protected vs. unprotected assemblages was found in the shallow infralittoral, demonstrating that, at least at local scale, protection can enhance community stability. Macrobenthos with long-lived and relatively slow-growing invertebrates and structurally complex algal forms were homogeneously distributed in space and went through little fluctuations in time. In contrast, a mosaic of disturbed patches featured unprotected locations, with small-scale shifts from macroalgal stands to barrens, and harsh temporal variations between the two states. Opposite patterns of spatial and temporal variability were found for the midlittoral assemblages. Despite an overall clear pattern of seagrass regression through time, protected meadows showed a significantly higher shoot density than unprotected ones, suggesting a higher resistance to local human activities. Our results support the assumption that the exclusion/management of human activities within MPAs enhance the stability of the structural components of protected marine systems, reverting or arresting threat-induced trajectories of change.

## Introduction

Over the past decades, both marine and terrestrial biodiversity experienced rapid global erosion [1]. Ensuing concern about the consequences of biodiversity loss fuelled theoretical and empirical studies aimed at unravelling the role of biodiversity in maintaining ecosystem properties and the goods and services they provide to humans [2]. Most research indicates that highly diverse assemblages increase the efficiency of ecosystem processes, being also less variable in space and time and more resistant to invasion and disturbance than low-diversity assemblages [1]–[3]. However, results are far from being unequivocal and the potential link between biodiversity and several ecosystem processes is still debated [4], [5]. Understanding if and how conservation and management actions, while restoring biodiversity, could have a role in maintaining the functional properties of marine and terrestrial ecosystems is therefore overriding [6].

Rising temporal and spatial variability is often a subtle outcome of human disturbance on ecological systems [7]–[9], undermining community structure, leading to decreased resilience and to increased potential for regime shifts [10]. Regime shifts are largely unpredictable due to the inherent complexity of ecological systems [11]. The spatial and temporal heterogeneity of a given system might shed light on determinants of its vulnerability and guide actions to mitigate the risk of critical transitions towards degraded states [12]. Changes in spatial and temporal variability may inform about the ongoing effects of natural or anthropogenic disturbance [13], [14], or serving as warning signals of approximating transitions [15].

Tropical coral reefs [16]–[18], temperate and boreal intertidal and subtidal rocky reefs [19], [20]–[22], and temperate coastal pelagic systems [23], [24] are all experiencing dramatic changes in populations, species, or entire functional groups leading to regime shifts that could be long-lasting or even irreversible [25]. The returning to previous undisturbed conditions is difficult, unless the major drivers of change, such as terrestrial runoffs, nutrient loading, pollution or fishing pressure and/or their destabilizing effects are reduced [26]. Also, while reverting from altered states, damaged marine ecosystems often follow different trajectories of recovery from that observed during decline [26].

Marine Protected Areas (MPAs) cannot be considered a global solution to biodiversity loss [27]. However, if properly managed, they can play a major role in reducing cumulative impacts [22], [28]–[30]. By excluding (or regulating) human activities, MPAs can enhance fisheries yields outside their boundaries through spill over [31], [32], promote biodiversity recovery with greater richness and abundance (or biomass) of species within protected conditions [33], [34], empower local communities and indirectly provide additional income from tourism [3].

These effects should also enhance the stability of single species and whole communities (i.e. the persistence in abundance and species composition through time) within MPAs, increasing their resistance (i.e. the ability to remain unperturbed despite the occurrence of a given disturbance) and/or resilience (i.e. the ability to absorb recurrent natural and human perturbations without slowly degrading or unexpectedly flipping into alternate states) and providing an insurance against the consequences of large-scale human threats. Variations in functional traits, identity, evenness and spatial distribution of species structuring communities might trigger different responses to disturbances in protected systems [35]. An effective protection, thus, may reduce the variability of ecological responses to natural and/or anthropogenic disturbance [30], [36], generating a ‘buffer effect’ that could minimize variations in density, biomass, and diversity of protected populations over time [37]. However, evidences of the role of MPAs in sustaining the stability of communities and ecosystems are scant [3], [38]–[40]. Theoretical work on stability has outpaced experimental work stressing the need for long-term experiments to assess temporal stability, as well as recovery from a variety of disturbances [4]. Decadal-scale observations of MPAs documented increased resilience [41], suggesting that the limited availability of long-term monitoring programs can be one of the reasons behind the general paucity of evidences on the stability of the effects of protection [42]. In addition, most studies focused on populations of single target species [40], [42] with few exceptions involving whole assemblages [43]. Attempts to document and compare changes in spatial and temporal variability of habitats and assemblages in response to protection regimes are extremely scant [44].

**Figure 1:**
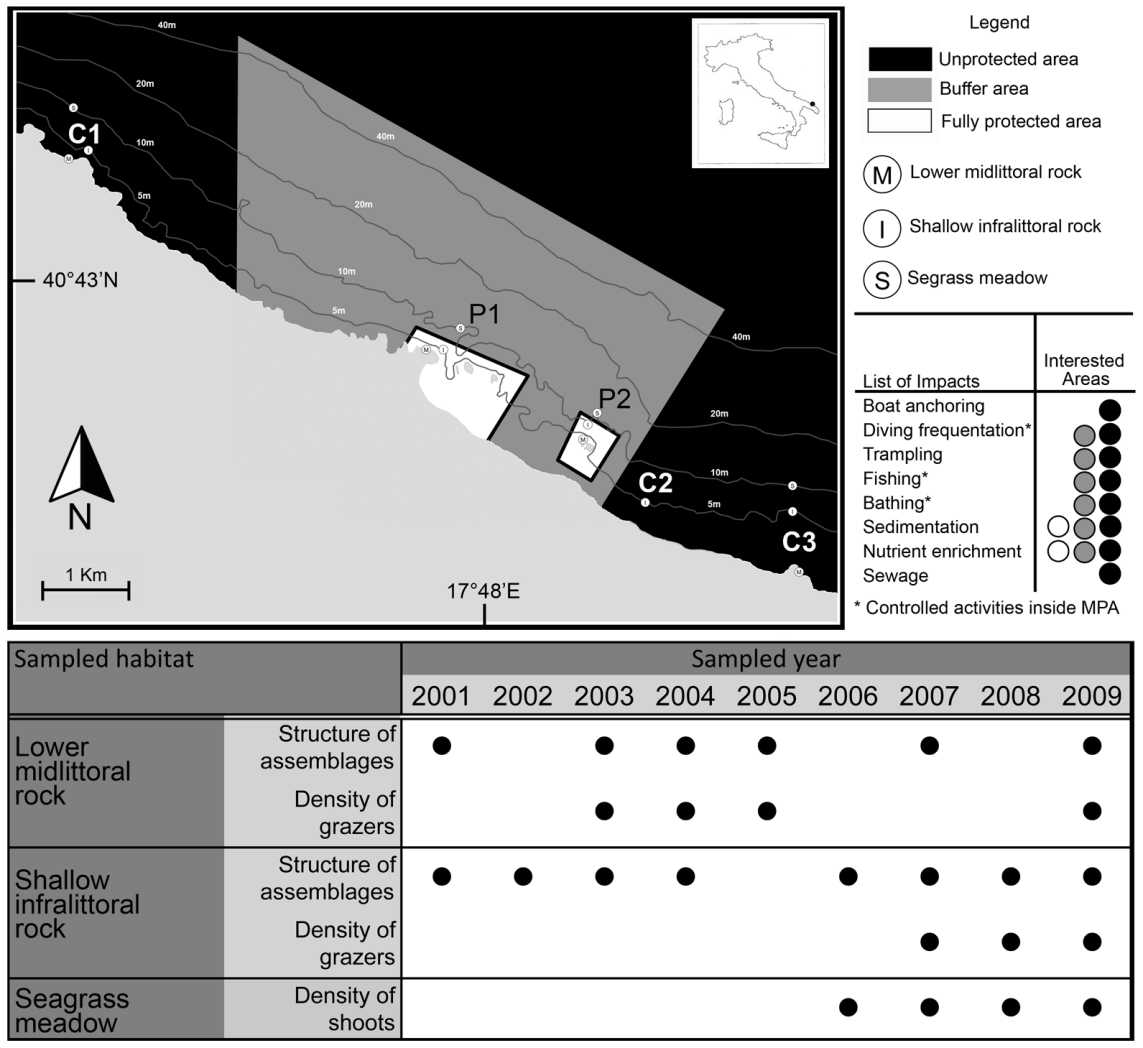
Framework of the study area and sampling timesheet. Map of the study area. No-take, no access areas are given in white, the buffer area of the MPA in grey, in black the unprotected area. P1, P2 = protected locations; C1, C2, C3 = unprotected locations. The main sources of human disturbance acting within and outside the MPA, along with the years in which sampling was carried out in each habitat are also provided (details in the legend).

Here, we use a nine-year data set to examine the potential of full protection regime of a Mediterranean MPA to affect the spatial and temporal variability of the assemblages inhabiting the rocky lower midlittoral (–0.1 to 0.1 m across mean-low-level water), the assemblages of the shallow infralittoral rocks (5–7 meter depth), and the *Posidonia oceanica* meadows (approximately 8–10 meter depth). Since its institution, outfall discharge and trawling are excluded from the whole MPA. Artisanal and recreational fishery, anchoring, trampling, diving frequentation, and maritime traffic are also severely regulated within the buffer area of the MPA and completely excluded from the two fully protected areas (Fig. 1). All these activities have been documented to directly and indirectly affect benthic habitats and assemblages [45]. Macroalgal canopies and seagrasses, in particular, can largely benefit from the exclusion of human activities. For example, predatory interactions are re-established when protection from exploitation is effective, causing the decrease of grazing pressure on rocky reefs [46]. The regulation of direct disturbances such as trawling, artisanal fishery, anchoring can halt the decrease in the shoot density and the ultimate regression of *P. oceanica* meadows [47]. In addition, the local control of the water quality can limit the shift to species with lower structural complexity such as turf forming, filamentous or other ephemeral seaweeds [21]. This study tests the hypothesis that the exclusion of human activities occurring within MPAs can be effective in reverting or halting threat-induced trajectories of change of *P. oceanica* meadows, of the rocky midlittoral and the shallow infralittoral assemblages.

## Materials and Methods

### Study Area

The study was carried out in the MPA of Torre Guaceto (40°42′N; 17°48′E), SE Italy (Adriatic Sea) (Fig. 1), covering a total surface of about 2.207 ha, consisting in two fully protected areas (i.e. no-take, no-access), and a buffer area, where few human activities are permitted and strictly regulated (Fig. 1). The MPA, instituted in 1991, owns an adequate enforcement [48] combined with a full involvement of fishermen in conservation and sustainable practices [49] that determined a significant recovery of target fish populations (mainly sparids and labrids) [34], [46]. A full description of habitats and assemblages present in the MPA is available in Fraschetti et al. [50], which also includes a bathymetric map of the whole MPA with the georeferred spatial information about the distribution and extent of habitats. The list of taxa found in the lower midlittoral and the shallow infralittoral rocks are reported in the Supporting Information S1.

All necessary permits were obtained from the Italian Ministry of the Environment and Protection of Land and Sea, and from the Direction of the MPA.

### Sampling Design

#### Benthic assemblages of the lower midlittoral rock

This is an algal-dominated assemblage mostly characterized by erect (e.g. *Corallina officinalis*, *Jania rubens*) and encrusting coralline algae (e.g. *Lithophyllum* spp.), *Laurencia* spp. and filamentous algae (e.g. Ceramiales) [50]. Sessile invertebrates, boring sponges (i.e. *Cliona* spp.) and anthozoans (e.g. *Actinia equina*) can be occasionally present. Sampling was carried out in six dates from 2001 to 2009 separated by at least one year at a depth comprised between - 0.1 to 0.1 m across mean-low-level water. Rocky substrates account for the 50% of the midlittoral of the MPA [50]. All sampling activities were carried out in the late spring-early summer to avoid possible effects of seasonality in the data. Samplings were undertaken at two locations within the no-take zones (P1 and P2) and at two unprotected locations (C1 and C3) outside the boundaries of the MPA (Fig. 1). Locations were positioned few kilometres from each other and were characterized by similar features in terms of substrate slope and wave exposure. Three sites (approximately 100–300 m apart) were randomly sampled at each location. At each site, ten 20×20 cm random quadrats were sampled to estimate *in situ* the abundance of sessile organisms. The division of each quadrat into 25 sub-quadrats eased visual estimates of the presence of taxa [51]. Final values were expressed as percentages of cover. Organisms that were not easily identifiable at species level were lumped into higher taxonomic groups or into morphological groups [52] (details are reported in the Supporting Information S1).

Due to the potential role of herbivores in structuring macroalgal assemblages [53], at each site, ten additional 20×20 cm random quadrats were used to count *in situ* the densities of dominant grazers (i.e. Polyplacophora, the gastropods of the genera *Patella* spp. and *Phorcus* spp.) in four sampling times, from 2003 to 2009, in the same locations and sites where sessile assemblages were also sampled.

#### Benthic community of the shallow infralittoral rock

This is an algal-dominated community at 5–7 m depth, where species such as *Flabellia petiolata*, *Halimeda tuna*, *Padina pavonica*, *Laurencia* spp. and the order of Dictyotales, dark filamentous algae (*Ectocarpus* spp. and *Sphacelaria* spp.), Cladophorales, encrusting coralline red algae (such as *Lithophyllum frondosum, L. incrustans, Mesophyllum alternans*) can be dominant. Sponges such as *Chondrilla nucula*, *Aplysina aerophoba*, *Ircinia variabilis*, *Cliona* spp. and *Phorbas* spp.) can be also present. In the infralittoral, rocky substrates account for the 10% of bottom surface within the MPA [50]. Sampling was conducted in eight random occasions from 2001 to 2009 (same season, i.e. late spring-early summer, as for midlittoral assemblages) at two protected (P1, P2) and two unprotected locations (C1, C3), with three sites in each location. A further unprotected location (C2) was also sampled from 2003 to 2006 (Fig. 1). The variable number of unprotected locations sampled during the study depends on the fact that the sampling program combines data coming from different projects funded for different periods. Assemblages were sampled photographically using a Nikonos V underwater camera, 28 mm focal length, close-up macro-system and two SB 105-Nikon electronic strobes. Thirteen random photographic sample of 16×23 cm were photographed at each site and 10 of them were randomly selected and analysed. This prevented the risk of having blurred, unclear photographic samples. In each photographic sample, the cover of sessile organisms was estimated under magnification by superimposing a transparent grid of 24 sub-quadrats on the entire photographed surface, and final values were expressed as a percentage. Destructive samples were collected for later identification of organisms present in the slides. Organisms not identified at species level were lumped into higher taxonomic groups or into morphological groups. Full taxonomic details are reported in the Supporting Information S1.

The sea urchins *Paracentrotus lividus* and *Arbacia lixula* are both present in the algal-dominated community of the infralittoral rocks of the MPA. Density of both species was estimated in three sampling dates (once per year from 2007 to 2009) in two protected locations (P1, P2) and two unprotected locations (C1, C3) where the sessile assemblages were also sampled (Fig. 1), with two sites for each location. At each site, underwater identification and counts of sea urchins within 20 replicate 1 m^2^ quadrats were performed in the infralittoral rocks. Counts were made at approximately 3–8 m depth during the daylight. Care was taken to search for urchins in crevices.

### Seagrass Meadows

*Posidonia oceanica* accounts for about the 20% of the infralittoral of the MPA [50]. Sampling was carried out in July, once per year, from 2006 to 2009. Due to the lack of seagrass beds within the no-take zones, the density of shoots was sampled in close proximity of P1 and P2 but in the buffer zone, where the seagrass forms extensive meadows [50] but human activities potentially affecting this habitat (i.e. anchoring, trawling) are also banned (see the Introduction and Fig. 1). Also in this case, two unprotected locations outside the MPA (C1 and C3) were sampled (Fig. 1). At each location, two patches (100–300 m apart) were randomly chosen within beds at 8–10 m depth. In each patch, the density of shoots was estimated *in situ* within five 1 m^2^ random quadrats. The status of *P. oceanica* beds was evaluated on the basis of the number of shoots per square meter following Pergent et al. [54].

### Statistical Analyses

#### Multivariate analyses

A distance-based permutational multivariate analysis of variance (PERMANOVA, [55]) was performed separately on the data sets relative to the lower midlittoral and shallow infralittoral rocky assemblages (sessile benthos only, grazers not included) to test for the effect of protection on their structural features (species composition and abundance). The analyses were based on Bray-Curtis dissimilarities calculated on untransformed data and each term was tested using 4999 random permutations [56].

For the analysis of the lower midlittoral rocky assemblages, the experimental design consisted of four factors: Time (T, 6 levels, random), Protection (P, 2 level, fixed), Location (L(P), 2 levels, random, nested in P) and Site (S(L(P)), 3 levels, random, nested in L(P)), with *n* = 10. For the shallow infralittoral rock community, the design and the factor labelling were the same but the number of levels differed for the factor T (8) and the number of sites and/or unprotected locations actually varied at different sampling times (see ‘Sampling design’ section for details). However, formal tests were still possible since PERMANOVA allows the handling of complex unbalanced designs [56]).

As the analysis of the lower midlittoral rocky assemblages showed significant temporal variations of differences among locations (see Results), a non-metric multidimensional scaling ordination (nMDS) of T × L(P) centroids was plotted to visualize patterns of variation among location through time. In the case of the shallow infralittoral, nMDS ordination of T × P centroids was plotted to visualize multivariate patterns of differences between protected and unprotected assemblages through time, since PERMANOVA revealed a significant effect of protection on temporal trajectories of assemblages (see Results). Centroids were obtained calculating principal coordinates (PCO) on the basis of the Bray–Curtis dissimilarity matrices among all pairs of units.

For the shallow infralittoral rocky assemblages, a canonical analysis of principal coordinates (CAP, [57]) was also performed for the T × P interaction term, calculating the distance matrix among sites in protected and unprotected locations in each time of sampling. Distinctness among T × P groups was assessed using leave one-out allocation success [58]. Individual taxa that might be responsible for any group differences seen in the CAP plot were investigated by calculating product–moment correlations of original variables (taxa) with canonical axes [57]. These correlations of individual variables with the two canonical axes (*r^1^* and *r^2^*) were then represented as lines in the CAP plot. Taxa were included in the plot only if exceeding an arbitrarily chosen value of correlation (i.e. 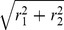) ≥ 0.2 [57].

#### Univariate analyses

ANOVA on multivariate estimates of spatial variability was employed to test for differences between protected and unprotected assemblages (sessile benthos only, grazers not included) at all investigated spatial scales (i.e. replicates, sites, locations), separately for the lower midlittoral and the shallow infralittoral rocky assemblages.

Components of variation from hierarchical analyses of variance allow separating sampling error from estimates of true variability associated to each spatial scale of observation [59]. However, a formal test requires multiple estimates of variance components, whereas, starting from a single set of data, only a single estimate is available for each source of variation. To deal with this issue, a single data set could be split into subsets allowing calculation of replicated independent estimates of variance components [60]. Each of the two data sets (i.e., lower midlittoral and shallow infralittoral rocky assemblages) was thus split into two halves, randomly selecting five replicates out of ten for each site in each time of sampling. The two subsets were then analysed running separate PERMANOVAs for each Time and Protection level, following the corresponding full hierarchical design. This allowed obtaining two independent components of pseudo-variance for each spatial scale (i.e. location, site, replicate) in each time of sampling for both protected and unprotected assemblages thus allowing to perform an ANOVA considering Time, Protection, and Scale as factors. For both communities, the design consisted of three factors: Time (T, 6 levels for the lower midlittoral and 8 levels for shallow infralittoral, random), Protection (P, 2 levels, fixed), Scale (Sc, 3 levels, fixed), with *n* = 2. For significant interaction terms involving the factor Protection, the Student Newman-Keuls (SNK) test was used for *post-hoc* pair-wise comparisons of spatial variability between protected and unprotected assemblages.

ANOVA on multivariate estimates of temporal variability was done to test the hypothesis that protection could increase the stability of sessile benthic assemblages. For the shallow infralittoral rocky assemblages, location C2 was excluded from the analysis because not available in all sampling occasions. For each data set, data were analysed separately for each of the twelve sites (i.e. the six protected sites and the six unprotected sites) using PERMANOVA, obtaining pseudo-variance components associated to factor Time. This allowed calculating six multivariate estimates of temporal variability for protected and unprotected assemblages. The design for the analysis consisted of only one factor, Protection (2 levels, fixed) with *n* = 6.

ANOVA was employed to test for differences in shoot density of *P. oceanica* beds between protected and unprotected locations. The analysis was done separately for each time of sampling. The design for the analyses consisted of three factors: Protection (P, 2 level, fixed), Bed (B(P), 2 levels, random, nested in P), Patch (Pa(B(P)), 2 levels, random, nested in B(P)), with *n* = 5.

ANOVA was also used to test for differences in density of midlittoral and shallow infralittoral grazers (i.e. gastropods and sea urchins respectively) between protected and unprotected locations. The analyses were done separately for each time of sampling. The three-factorial design for analyses consisted of factor Protection (P, 2 level, fixed), Location (L(P), 2 levels, random, nested in P), Site (S(L(P)), 3 levels for the lower midlittoral and 2 levels for the infralittoral grazers, random, nested in L(P)), with *n* = 10 for the lower midlittoral and *n* = 20 for the shallow infralittoral.

Prior to all ANOVAs the homogeneity of variance was examined using the Cochran’s *C* test. All analyses were performed using the GMAV 5 software (University of Sydney, Australia).

## Results

### Benthic Assemblages of the Lower Midlittoral

**Figure 2:**
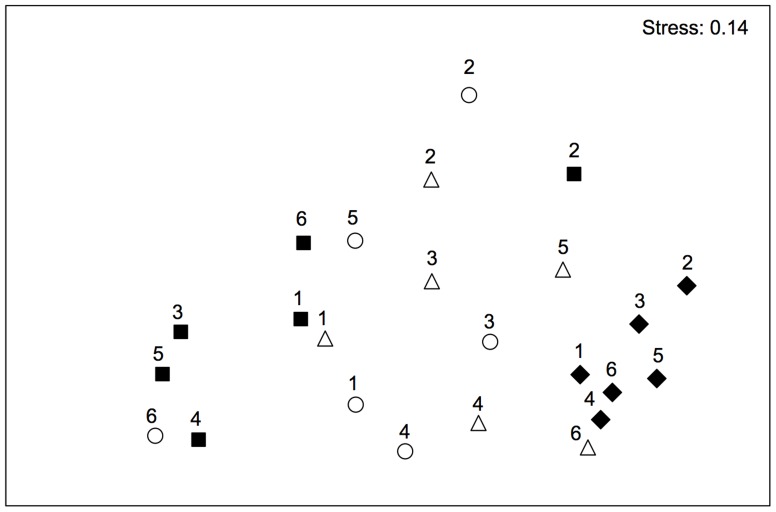
Differences in the multivariate structure of midlittoral assemblages. Non-metric multidimensional scaling ordinations (nMDS) of T × L(P) centroids based on Bray-Curtis dissimilarity measures for the midlittoral assemblages (○ = P1; ▵ = P2; ▪ = C1; ⧫ = C2; P = protected locations; C = unprotected locations). Numbers (from 1 to 6) indicate the sampling times.

Multivariate analyses showed no effects of protection on the structure of assemblages (Table 1). After more than 20 years from its institution, inside and outside the MPA, benthic assemblages were largely dominated by erect and encrusting coralline algae, few boring sponges and anthozoans [50]. Results of PERMANOVA showed significant T × L(P) and T × S(L(P)) interactions, indicating a significant assemblage variability among locations, and among sites within locations, that varied in time (Table 1). In other words, despite similarities in taxa composition were found between assemblages from protected and unprotected locations, their patterns of distribution differed in space and time. Temporal variations among locations were portrayed in the nMDS ordination of T × L(P) centroids (Fig. 2), which also showed a higher scattering of centroids of protected locations with respect to unprotected locations, suggesting higher temporal variations in protected assemblages than in unprotected ones. An exception to the observed patterns was detected especially in Time 2, where one of the unprotected locations was featured by particularly high abundances of articulated corallines, such as *Corallina* and *Jania*.

**Table 1:** Summary of PERMANOVAs testing for the effect of protection on the lower midlittoral and on the shallow infralittoral rocky assemblages.

	Lower midlittoral			Shallow infralittoral		
Source ofvariation	MS	*F*	*P*	MS	*F*	*P*
Time = T	42693.0	2.71	**0.003**	38853.0		
Protection = P	17534.0	0.30	0.999	50270.0		
Location(P) = L(P)	91908.0	4.09	**0.000**	25098.0		
Site(L(P)) = S(L(P))	8200.5	1.34	0.101	5704.7		
T × P	17235.0	1.09	0.375	18169.0	2.31	**0.000**
T × L(P)	15734.0	2.58	**0.000**	7969.8	2.32	**0.000**
T × S(L(P))	6098.2	5.44	**0.000**	3460.6	2.80	**0.000**
Residuals	1121.5				1235.8	

*P*-values are given in bold (see text for details). Analyses were based on Bray-Curtis dissimilarities and each test was performed using 4999 permutations of appropriate units. Only tests for the terms relevant to hypothesis have been reported. Significant

**Figure 3:**
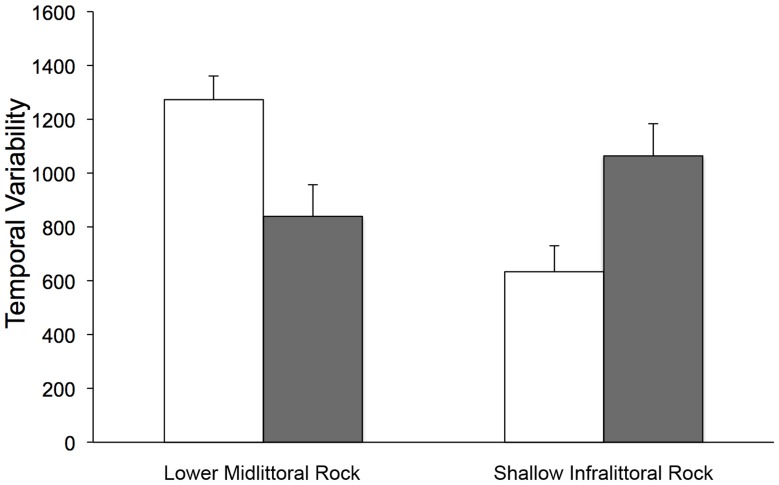
Temporal variability of rocky bottom assemblages. Mean temporal variability (i.e. estimates of variance associated to factor Time, see methods for further details) ± SE (*n* = 6) of the lower midlittoral and the shallow infralittoral rocky assemblages. White bars = protected assemblages; grey bars = unprotected assemblages.

Results of ANOVA on multivariate estimates of spatial variability of assemblages showed a significant T × P × Sc interaction, indicating an effect of protection on spatial heterogeneity of assemblages, varying in time and with scale (Table 2). *Post-hoc* pair-wise comparisons indicated an increase through time of spatial heterogeneity in protected assemblages at the scale of replicates (10 s of centimetres) and locations (kilometres), but not at the scale of sites (100 s of metres) (Table 2). ANOVA on estimates of multivariate temporal variability detected a significant effect of protection (*F* = 8.75, *P*<0.05), with a higher temporal variability of protected assemblages with respect to unprotected ones (Fig. 3). In other words, protected assemblages were more spatially heterogeneous and with more pronounced temporal fluctuations than the assemblages outside the MPA.

**Table 2:** Summary of ANOVAs testing for the effect of protection on multivariate estimates of spatial variability for the lower midlittoral and the shallow infralittoral rocky assemblages.

	Lower midlittoral				Shallow infralittoral					
Source ofVariation	MS		*F*	*P*	MS			*F*		*P*
Time = T	381348.4		6.81		369939.5			6.10		
Protection = P	254713.6		4.55		834647.2			13.76		**0.000**
Scale = Sc	705000.6		12.58		7345335.6			121.11		
T × P	180268.2		3.22		108618.0			1.79		0.111
T × Sc	539617.6		9.63		136143.5			2.24		**0.019**
P × Sc	1620263.0		28.92		126932.8			2.09		0.134
T × P × Sc	131370.3		2.34	**0.030**	77770.6			1.28		0.253
Residuals	56022.6							60651.4		
**SNK Lower** **midlittoral**										
	**Time**									
**Scale**	**1**	**2**		**3**		**4**	**5**		**6**	
Location	P<Cs	P<Cs		P<Cs		P<Cs	P<Cs		P = Cs	
Site	P>Cs	P = Cs		P>Cs		P = Cs	P = Cs		P>Cs	
Replicate	P = Cs	P = Cs		P = Cs		P = Cs	P = Cs		P>Cs	

= Protected locations; Cs = Unprotected locations. Terms already involved in significant higher-order interactions were not reported. Significant *P*-values are given in bold. SNK pair-wise tests for significant interaction terms involving the factor Protection (i.e. only T × P × Sc, in the lower midlittoral rocks) are given below in the table. P

**Figure 4:**
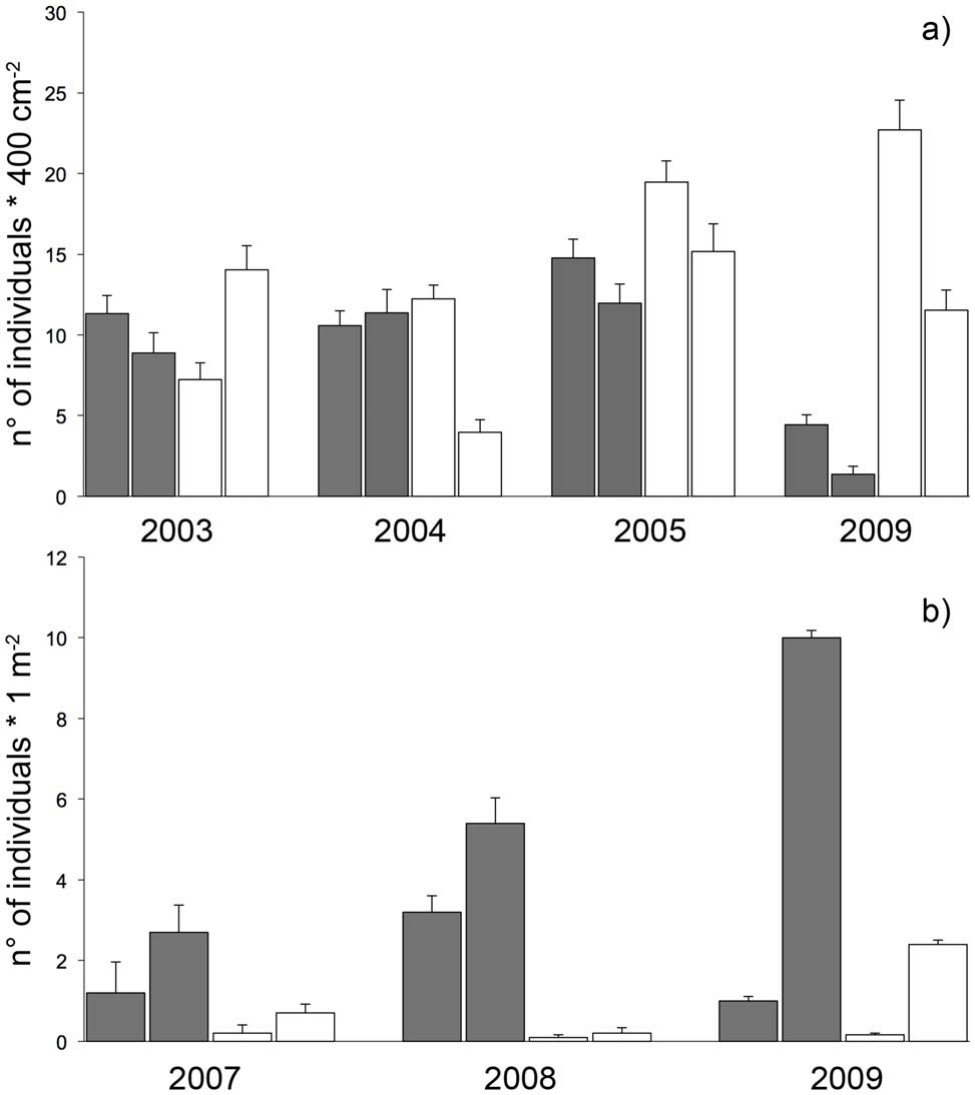
Relative amount of grazers on both rocky habitats. Mean density (± SE) of grazers found in the (a) lower midlittoral (gastropods) and in the (b) shallow infralittoral (sea urchins) rocky assemblages in each time of sampling. White bars = protected locations; grey bars = unprotected locations.

ANOVA on density of grazers (i.e. *Patella* spp. and *Phorcus* spp.) did not highlight a significant effect of protection through time (Table 3). However, inspection of the graph in Fig. 4a suggested a decrease of grazers in the unprotected locations and, on average, an increase in protected locations in time. More particularly, in 2009 (Time 4), the density of these grazers was five times higher in protected locations than in unprotected ones, although such differences were not significant probably due to the high variability at the scale of sites.

**Table 3:** Summary of ANOVAs testing for the effect of protection on the total abundance of midlittoral grazers in each sampling time.

	2003			2004			2005			2009		
Source ofvariation	MS	*F*	*P*	MS	*F*	*P*	MS	*F*	*P*	MS	*F*	*P*
Protection = P	8.5	0.02	0.896	246.5	0.48	0.561	468.1	2.37	0.263	6063.4	6.03	0.133
Location(P) = L(P)	392.4	2.20	0.173	517.3	8.46	**0.011**	197.5	3.68	0.074	1005.7	14.91	**0.002**
Site(L(P)) = S(L(P))	178.1	4.84	**0.000**	61.2	2.02	0.050	53.7	0.47	0.472	67.4	1.68	0.112
Residuals	36.8			30.2			56.0			40.2		

*P*-values are given in bold. Significant

### Benthic Assemblages of the Shallow Infralittoral Rock

**Figure 5:**
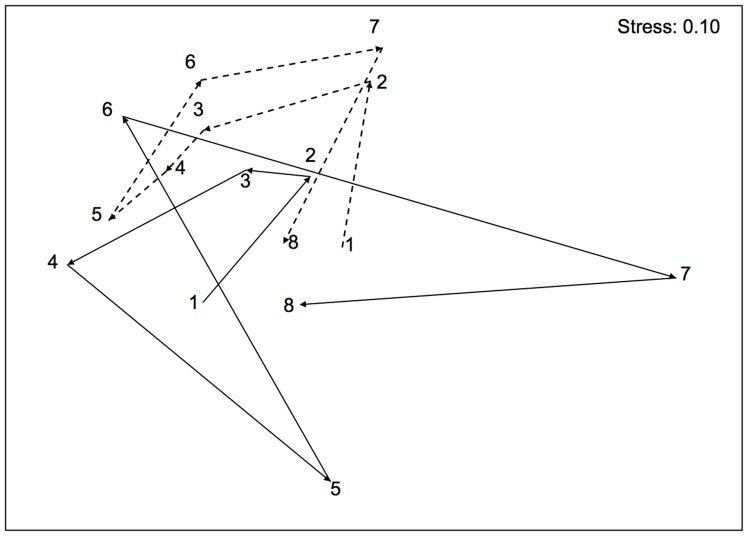
Differences in the multivariate structure of the shallow infralittoral rocky assemblages. Non-metric multidimensional scaling ordinations (nMDS) of T × P centroids based on Bray-Curtis dissimilarity measures. Dotted trajectories = protected assemblages; solid trajectories = unprotected assemblages. Numbers (from 1 to 8) indicate the sampling times.

A significant effect of protection in modifying temporal trajectories of sessile assemblages was detected by PERMANOVA, as indicated by the significant T × P interaction (Table 1). These differences in temporal trajectories between protected *vs.* unprotected assemblages were clearly portrayed in the nMDS ordination plot of T × P centroids, which also underlined more severe temporal changes of unprotected *vs*. protected assemblages (Fig. 5). In Time 1, protected and unprotected assemblages were quite close to each other and showed similar patterns of temporal variation until Time 3, whereas, starting from Time 4, their trajectories diverged following different directions that led to a clear separation of centroids in Time 5 and 7, coming very close again at Time 8.

**Figure 6:**
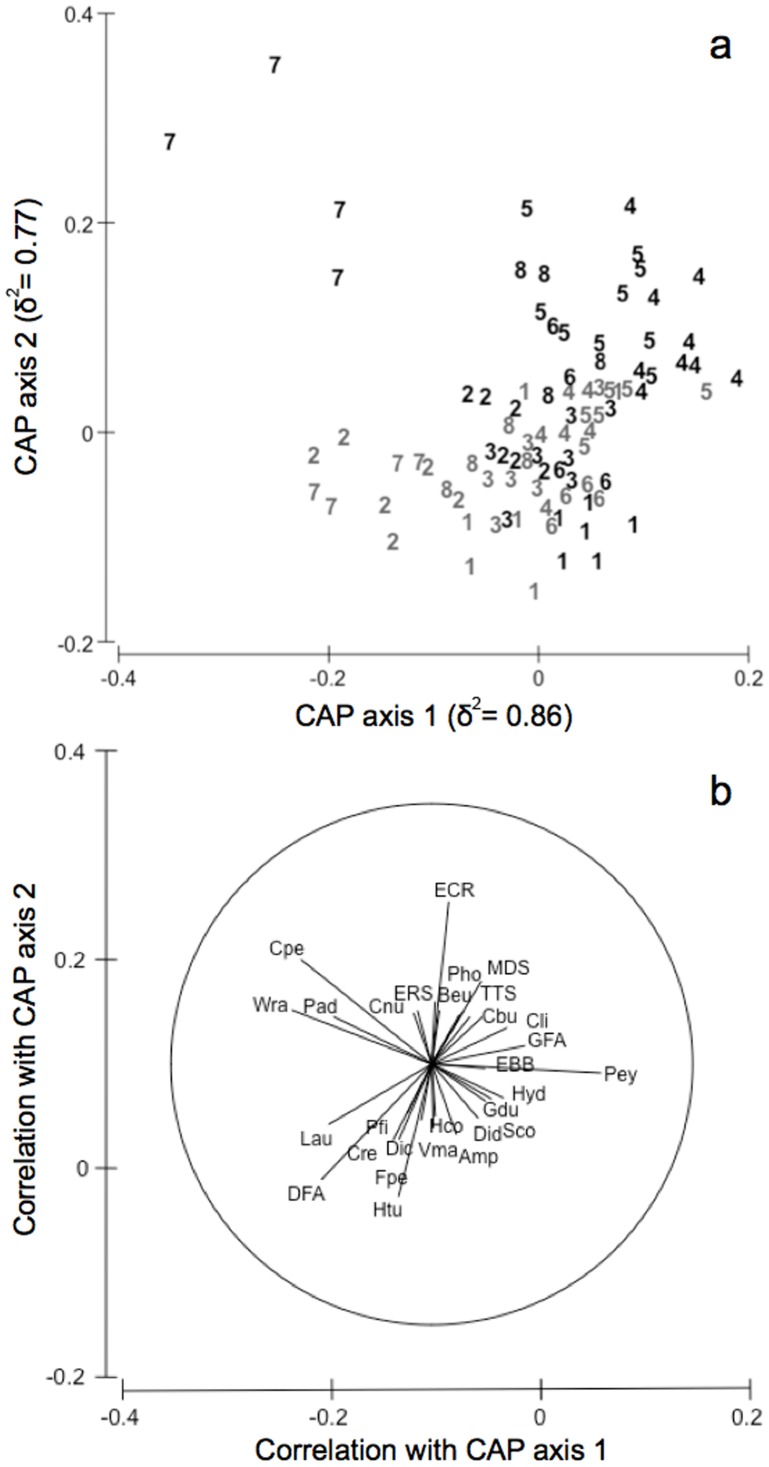
Canonical analysis of principal coordinates for protected *vs.* unprotected shallow infralittoral rocky assemblages and discriminating taxa. CAP for factor T × P based on the distance matrix of sites of the shallow infralittoral rocky assemblages (a). Numbers (from 1 to 8) indicate progressive times of sampling. Grey numbers = protected assemblages; black numbers = unprotected assemblages. Individual taxa highly correlated with canonical axes were also shown (b) (see Supporting Information S1 for taxa abbreviations).

The canonical analysis of principal coordinates for the term T × P achieved the highest allocation success (52.75%) using *m* = 9 principal coordinate (PCO) axes, which explained 90.1% of variation in the original dissimilarity matrix. The two canonical axes had very high canonical correlations with the multivariate assemblages (δ^2^, Fig. 6a, *P = *0.001). The CAP analysis revealed that harsh temporal changes outside the MPA corresponded to alternate states of assemblage structure whereas, within the MPA, assemblage structure remained relatively homogeneous through time (Fig. 6). Starting from Time 1, unprotected assemblages move counter clockwise from the down right corner of the graph, reaching the up left corner (Time 7) and coming back on the up right side (Time 8) (Fig. 6a), shifting from assemblages characterized by erect-canopy [*Sphaerococcus coronopifolius* (Sco)], turf-forming [*Amphiroa* spp. (Amp), *Valonia macrophysa* (Vma)] algae, and several invertebrates [*Hemimycale columella* (Hco), Didemnidae (Did), Hydroids (Hyd), *Gastrochaena dubia* (Gdu)] (Time 1, 2, 3), to assemblages mostly characterized by encrusting [Encrusting Calcified Rhodophytes (ECR), *Peyssonnelia* spp. (Pey)], ephemeral [Green Filamentous Algae (GFA)] algae, encrusting invertebrates [e.g., Encrusting Brown Bryozoans (EBB), *Balanophyllia europaea* (Beu), Encrusting Red Sponges (ERS), *Chondrilla nucula* (Cnu)] and boring sponges [Clionidae (Cli)] (Time 4, 5, 6, 8), passing by assemblages dominated by turf-forming algae [*Wrangelia penicillata* (Wra), *Padina pavonica* (Pad)] and the anthozoan *Cereus pedunculatus* (Cpe) (Time 7) (Fig. 6b). In contrast, protected assemblages in all times clustered together on the down side of the graph (Fig. 6a) being distinguished by taxonomic groups of erect-canopy [*Dictyota* spp. (Dic), *Laurencia* complex (Lau), *Sphaerococcus coronopifolius* (Sco)], turf-forming [*Flabellia petiolata* (Fpe), *Amphiroa* spp. (Amp), *Halimeda tuna* (Htu), Dark Filamentous Algae (DFA)] algae, sponges [*Petrosia ficiformis* (Pfi), *Chondrosia reniformis* (Cre), *Hemimycale columella* (Hco),] and other invertebrates [Didemnidae (Did), Hydroids (Hyd), *Gastrochaena dubia* (Gdu)] (Fig. 6b).

Results of ANOVA on multivariate estimates of spatial variability of assemblages detected significant differences between protected and unprotected locations that were consistent in time and across scales (Table 2), indicating a significant effect of protection in decreasing spatial heterogeneity of assemblages at the scale of tens of centimetres, among replicate units, up to kilometres (results not showed). In other words, long-lived and relatively slow-growing invertebrates and structurally complex algal forms were homogeneously distributed in space and were featured by less fluctuations in time compared to unprotected assemblages.

The analysis on estimates of multivariate temporal variability also detected a significant effect of protection (*F* = 4.99, *P*<0.05) with a lower value in protected than in unprotected assemblages (Fig. 3), indicating an effect of protection in smoothing out the temporal variability of assemblages.

Inspection of graph in Figure 4b suggested an increase of grazers (i.e. sea urchins) in unprotected locations through time. However, ANOVA did not highlight a significant effect of protection on their density. Also, a significant variability in their pattern of distribution was documented at the scale of sites in all sampling times (Table 4).

**Table 4:** Summary of ANOVAs testing for the effect of protection on the total abundance of sea urchins of the shallow infralittoral in each time of sampling.

	2007			2008			2009		
Source of variation	MS	*F*	*P*	MS	*F*	*P*	MS	*F*	*P*
Protection = P	2.8	0.69	0.494	688.9	15.48	0.059	57.6	0.96	0.431
Location(P) = L(P)	4.0	1.68	0.295	44.5	0.47	0.655	60.1	6.08	0.061
Site(L(P)) = S(L(P))	2.4	6.02	**0.002**	94.5	4.95	**0.001**	9.9	4.39	**0.002**
Residuals	0.1			19.1			2.2		

*P*-values are given in bold. Significant

### Seagrass Meadows

**Figure 7:**
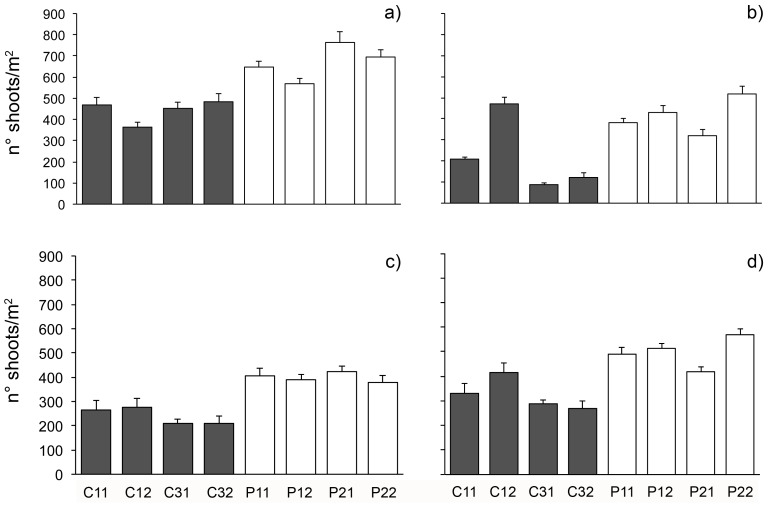
Variations of seagrass shoot density in the different locations. Mean (± SE, *n* = 5) n° of shoots/m^2^ of *P. oceanica* in each patch in (a) 2006, (b) 2007, (c) 2008 and (d) 2009. White bars = protected beds; grey bars = unprotected beds.

Results of ANOVA on shoot density (Table 5) showed an overall lack of significant differences between protected and unprotected *P. oceanica* seagrass beds (Fig. 7). Inspection of graphs in Figure 7 suggested a general reduction of shoot density through time that was lower in protected than in unprotected beds. Even if the pattern is quite clear, in one of unprotected areas, the number of shoots increased in 2007, reaching densities comparable to protected areas. The classification of the status of *P. oceanica* beds following Pergent et al. [54] revealed that, in contrast to beds within the MPA, which were characterized by general undisturbed conditions in all times, unprotected beds showed a progressive shift from undisturbed towards disturbed or very disturbed conditions (Table 6).

**Table 5:** Summary of ANOVAs testing for the effect of protection on the shoot density of *P. oceanica* beds in each sampling time.

	2006			2007			2008			2009		
Source of variation	MS	*F*	*P*	MS	*F*	*P*	MS	*F*	*P*	MS	*F*	*P*
Protection = P	513022.5	11.82	0.075	361000.0	2.60	0.248	253446.4	27.35	**0.035**	292410.0	13.02	0.070
Beds(P) = B(P)	43397.0	3.03	0.375	138707.2	1.99	0.251	9267.2	6.21	0.059	22454.8	1.17	0.397
Patch(B(P)) = Pa(B(P))	14337.1	2.49	0.063	69535.2	19.30	**0.000**	1492.8	0.35	0.839	19122.0	5.00	**0.003**
Residuals	5765.6			3602.2			4215.8			3825.0		

*P*-values are given in bold. Significant

**Table 6:** Classification of the status of *P. oceanica* beds based on shoot density following Pergent et al. [54].

Location	Patch	2006	2007	2008	2009
P1	1	undisturbed	disturbed	Undisturbed	undisturbed
P1	2	undisturbed	undisturbed	Undisturbed	undisturbed
P2	1	undisturbed	disturbed	Undisturbed	undisturbed
P2	2	undisturbed	undisturbed	Undisturbed	undisturbed
C1	1	undisturbed	verydisturbed	verydisturbed	disturbed
C1	2	undisturbed	verydisturbed	verydisturbed	undisturbed
C3	1	disturbed	undisturbed	Disturbed	Disturbed
C3	2	undisturbed	verydisturbed	verydisturbed	Disturbed

= Protected locations; C1, C3 = Unprotected locations. The state of the seagrass beds has been reported for each investigated patch in each time (see text for details). P1, P2

## Discussion

Our case study supports the recent findings on the efficacy of MPAs, documenting further ecological consequences of reserve implementation. These results show that the effective protection of this area, while restoring biodiversity, can enhance assemblage stability, by increasing the spatial homogeneity of protected assemblages, decreasing their temporal variability and increasing their resistance to large-scale disturbance. After more than 20 years from the institution of the MPA, the presence of highly performing species with the re-establishment of continuous and persistent macroalgal canopies, and the maintenance of more dense and healthy meadows are an important outcome of a successful regulation of human activities. Our study contributes to demonstrate that reducing the effects of local sources of disturbance by excluding direct human activities within MPAs may represent an effective strategy in mitigating also the effects of large-scale threats [40]. Even though the MPA is embedded within a landscape featured by a low population density and coastal development, the whole area has been recently ranked as under the pressure of moderate to high human impact [61]: fifty-five fishermen are active in the area, outside the MPA, an industrial/commercial harbour with a cargo tonnage of 10 million tons and a sewage treatment for 130,000 inhabitants are at a distance of about 15 kilometres from the MPA. Results showing an effective mitigation from threats are clear for seagrass meadows and for assemblages from the shallow infralittoral, while different patterns have been found for midlittoral assemblages.

In the shallow infralittoral rocks, at fully protected locations, the exclusion of fishery, anchoring and diving frequentation had important consequences at assemblage level. Within the MPA, macrobenthic assemblages with long-lived and relatively slow-growing invertebrates (e.g. sponges, madreporarians) and structurally complex algal forms (i.e. erect-canopy forming algae like Dictyotales), were homogeneously distributed in space and went through little fluctuations in time. In contrast, a mosaic of disturbed patches featured unprotected locations, with small-scale shifts from macroalgal stands to barrens, and harsh temporal variations between the two states and an increase of sea urchins through time. Such differences in the structure of assemblages determined the different patterns of spatio-temporal variability between protected and unprotected areas (Fig. 3). Canopy-forming species create a complex three-dimensional habitat and can increase community stability by reducing the variability of environmental factors [62], [63], even though this effect could be context dependent [64], [65]. The effects of mechanical disturbance such as anchoring on erect algae have been largely demonstrated [66]. There are also strong evidences that the exclusion of fishery can re-establish lost predatory interactions, decreasing the density of sea urchins and enhancing top-down mechanisms able to drive the recovery of macroalgae in MPAs through cascade effects [34], [39], [46], [67]. Within an MPA, moreover, predators might dampen high-recruitment episodes of sea urchins, stabilizing their populations [42]. Such indirect effects may lead to an increased resilience of protected benthic assemblages by accelerating the processes of colonization after disturbance [43]. As also documented in previous studies [46], during the last decade, our results show that sea urchins were highly variable in abundance at unprotected locations and more stable and less abundant at protected locations. These patterns suggest that, in the absence of the selecting sea urchin removal by fish predators under protection regimes, fluctuating levels of biological disturbance on macroalgae determined by sea urchin grazing may be also responsible of the higher spatial and temporal variability in unprotected areas. Such findings reinforce the assumption that increased variability in space and time can be considered an indicator of (natural and/or anthropogenic) stress in benthic assemblages [14], [68]–[70] and support the existence of positive feedback related to dense macroalgal canopies on assemblage resistance potential.

Opposite patterns of spatial and temporal variability were found for the lower midlittoral rocky assemblages, with no effect of protection detected on their multivariate structure, largely dominated by articulate corallines, *Laurencia* and other Ceramiales inside and outside the MPA. Higher temporal variations and a general increase through time of spatial heterogeneity of assemblages within the MPA were also found. Interestingly, in protected locations (where the collection of organisms is forbidden), sea urchin populations of the shallow infralittoral rock decreased, whereas an increase in gastropod grazers was observed in the midlittoral. Idiosyncratic responses to protection among habitats and ecological compartments have been recently found also in others MPAs [69], [71]. While in the infralittoral the re-establishment of predatory interactions led to a control of grazing pressure, in the lower midlittoral, the exclusion of human collection of grazers such as *Patella* spp. and *Phorcus* spp. within no-take areas had a clear effect on their abundances and was possibly responsible for the general increase through time of the spatial heterogeneity in protected assemblages at all investigated spatial scales [72]–[74].

Direct effects of protection were observed also on the seagrass *P. oceanica*. Recently, a combination of available aerial photographs and results from the report of the European project EUROSION (http://www.eurosion.org) showed that the recent evolution of the littoral (ratio between the length of the stretches of coast in recession and the total coastal length) is towards a clear regression, probably causing the high sediment load observed in the whole area [75], which, in turn, can result in increased stress levels for seagrass meadows [76], [77]. Although an overall clear pattern of regression was observed in the whole region, a significantly higher shoot density characterized protected meadows with respect to unprotected ones. Such a pattern was consistent through time and suggests a higher resistance to coastal pressures of the protected meadows. In this case, the local management of fishing and anchoring inside the MPA seems to provide some insurance against large-scale pressures, such as sedimentation, which are impractical to manage directly. Montefalcone et al. [78] conclude that MPAs alone are not sufficient to guarantee the protection of *P. oceanica* meadows and that management tools other than MPAs are needed to enhance the large-scale persistence of this habitat [79], [80]. Activities that reduce coastal pollution and eutrophication by the establishment of management plans for water resources, for instance, are also key elements for the recovery of habitats such as *Cystoseira* spp. fringes [81]–[83]. Clear indications of thresholds and variables involved in these observed recovery trajectories have also been documented [84]–[86] and, at least for *P. oceanica*, it is apparent that the management of direct disturbances, such as trawling, anchoring, dredging and pipeline refilling, can help its restoration, even though over long time scales.

Natural fluctuations in abundance of species in space and time are extremely common in the marine environment [87] and deriving the conclusion that the protection of this area is the only process driving the observed changes is not realistic. Multiple processes acting simultaneously have the potential to interact causing changes that are difficult to interpret. The exceptions observed in our results (see Fig. 2 for the lower midlittoral rocks and the high shoot density in *P. oceanica* in one unprotected location) represent clear evidence that detailed information on the environmental context might have largely improved our potential to understand the variability in the observed outcomes. Even with these limits, our results suggest that the reduction of human activities, and especially overexploitation, can have clear positive consequences at habitat scale [88], but also that the ensuing direct effects on target populations can affect indirectly non-target species. A major limit to the understanding of interacting outcomes of protection and natural or anthropogenic disturbance on marine ecosystems relies on the scarcity of long-term studies. Having long-term funding to support studies as this one is still a challenge and this analysis represents an effort to ensure data comparability despite the lack of funding continuity. A long-term perspective in assessing the effects of protection is critical since different ecological components may respond differently and over varying temporal scales [41]. In addition, a deeper insight on causal processes underlying the dynamic responses of communities could not disregard their spatial and temporal heterogeneity.

The present study represents one of the first attempts in this direction. Even though we recognize that other processes might be involved in explaining the observed changes, by combining decadal time series of data on different habitats and assemblages, we reinforced the view that MPAs might provide an insurance against the consequences of local and large-scale human disturbances promoting the persistence of desirable ecological conditions and enhancing the stability of marine communities. Under future scenarios of frequent and/or persistent disturbance, conservation strategies based on MPA networks aimed at enhancing resilience may be the most effective tool to limit the negative impacts of the complex suites of threats on marine ecosystems.

## Supporting Information

Supporting Information S1 **Taxonomic list of the species (or species groups) recorded.**
(DOC)
