# Supplementary material for: Protection Enhances Community and Habitat Stability: Evidence from a Mediterranean Marine Protected Area
Source: PLoS One. 2013 Dec 11;8(12):e81838. doi: 10.1371/journal.pone.0081838 (PMC3859515; doi:10.1371/journal.pone.0081838)
Supplement: Supporting Information S1 — Taxonomic list of the species (or species groups) recorded. (DOC) [file pone.0081838.s001.doc]

**Supporting Information S1**

Taxonomic list of the species (or species groups) recorded. Acronyms for the CAP analysis are given in square brackets. I = Taxa found on intertidal rocky assemblages; S = Taxa found on shallow subtidal rocky assemblages.

# Taxa Grouping

# Prokaryotes^I^

# Rhodophyceae

Articulated Corallines [AC]^I,S^

*Jania rubens* (Linnaeus) J.V. Lamuroux 1812

*Haliptilon virgatum* (Zanardini) Garbary & Johansen

*Amphiroa* spp. [Amp] ^I,S^

*A. beauvoisii* J.V. Lamouroux 1816

*A. cryptarthrodia* Zanardini, 1843

*A. rigida* J.V. Lamouroux, 1816

*Botryocladia* sp.^S^

*Callithamnion* sp.^I^

*Champia parvula* (C. Agardh) Harvey^S^

*Chrysymenia ventricosa* (J.V. Lamouroux) J. Agardh, 1876^S^

*Corallina* spp.^I,S^

*C. elongata* Ellis & Solander, 1786

*C. officinalis* Linnaeus, 1758

Dumontiaceae^S^

*Dudresnaya verticillata* (Withering) Le Jolis, 1863

*Acrosymphyton purpuriferum* (J. Agardh) Sjöstedt

Encrusting Calcified Rhodophyceae [ECR]^I,S^

*Lithophyllum* spp.

*Lithothamnion* spp.

*Neogoniolithon* sp.

*Gastroclonium* sp.^I^

Gelidiaceae^I,S^

*Gelidium* spp.

*Pterocladiella* sp.

*Hypnea musciformis* (Wulfen) J.V. Lamouroux, 1813^I,S^

*Laurencia* complex [Lau]^I,S^

*Chondrophycus papillosus* (C. Agardh) Garbary & J. Harper

*Laurencia obtusa* (Hudson) Lamouroux, 1813

*Osmundea* sp.

*Liagora viscida* (Forsskål) C. Agardh, 1822^I^

*Peyssonnelia* spp. [Pey]^I,S^

*P. dubyi* P.L. Crouan & H.M. Crouan, 1844

*P. harveyana* P.L. & H.M. Crouan ex J. Agardh

*P. polymorpha* (Zanardini) F. Schmitz

*P. rubra* (Greville) J. Agardh, 1851

*P. squamaria* (S.G. Gmelin) Decaisne, 1839

*Sphaerococcus coronopifolius* Stackhouse, 1797^S^

*Tricleocarpa fragilis* (Linnaeus) Huismans

& R.A. Townsend, 1993^S^

*Wrangelia penicillata* (C. Agardh) C. Agardh, 1828 [Wra]^I,S^

# Phaeophyceae

*Colpomenia sinuosa* (Mertens ex Roth) Derbès & Solier, 1851^I,S^

Cutleriaceae^S^

*Cutleria adspersa* (Roth) De Notaris

*Zanardinia typus* (Nardo) P.C. Silva

*Cystoseira amentacea* Bory^I^

*Cystoseira compressa* (Esper) Gerloff & Nizamuddin, 1975^I^

*Dictyota* spp. [Dic]^I,S^

*D. dichotoma* (Hudson) J.V. Lamouroux, 1809

*D. spiralis* Montagne, 1846

*Padina pavonica* (Linnaeus) Thivy, 1960 [Pad]^I,S^

Stypocaulaceae^I,S^

*Halopteris filicina* (Grateloup) Kützing, 1843

*Stypocaulon scoparium* (Linnaeus) Kützing, 1843

# Chlorophyceae

*Acetabularia (Acetabularia) acetabulum* (Linnaeus) P.C.Silva, 1952^I,S^

*Anadyomene stellata* (Wulfen) C. Agardh, 1822^I,S^

*Caulerpa racemosa* (Forsskål) J. Agardh, 1873^S^

*Codium bursa* (Linnaeus) C. Agardh, 1822^S^

*Dasycladus* vermicularis (Scopoli) Krasser, 1898^S^

*Flabellia petiolata* (Turra) Nizamuddin [Fpe]^S^

Green Filamentous Algae^I,S^

*Bryopsis* spp.

*Chaetomorpha* spp.

*Cladophora* spp.

*Enteromorpha* spp.

*Halimeda Halimeda tuna* (Ellis & Solander)

J.V. Lamouroux, 1816 [Htu]^I,S^

*Palmophyllum crassum* (Naccari) Rabenhorst^S^

Ulvaceae^I^

*Valonia macrophysa* Kützing, 1843^I,S^

# Other algal groups

Dark Filamentous Algae [DFA]^I,S^

Ceramiales

Gigartinales

*Ectocarpus* spp.

*Sphacelaria* spp.

Thin Tubular Sheet-like algae^S^

*Rhodymenia* sp.

*Nitophyllum* sp.

Coarsely Branched algae^I,S^

*Chondria* sp.

*Gulsonia* sp.

# Porifera

*Agelas* sp.^S^

*Aplysina aerophoba* Nardo, 1843^S^

*Chlathrina* sp.^S^

*Chondrilla nucula* Schmidt, 1862^S^

*Chondrosia reniformis* Nardo, 1847^S^

*Cliona celata* Grant, 1826^I,S^

*Cliona* spp. [Cli]^I,S^

*C. viridis* (Schmidt, 1862)

*C. rhodensis* Rützler & Bromley, 1981

*Pione vastifica* (Hancock, 1849)

*Dysidea avara* (Schmidt, 1862)^I,S^

Encrusting Red Sponges [ERS]^I,S^

*Crambe crambe* (Schmidt, 1862)

*Spirastrella cunctatrix* Schmidt, 1868

*Hemimycale columella* (Bowerbank, 1874)^S^

*Ircinia variabilis* (Schmidt, 1862)^S^

Massive Black Sponges^I,S^

*Cacospongia* sp.

*Sarcotragus spinosulus* Schmidt, 1862

*Oscarella* sp.^S^

*Petrosia (Petrosia) ficiformis* (Poiret, 1789)^S^

*Phorbas fictitius* (Bowerback, 1866)^S^

*Terpios fugax* Duchassaing & Michelotti, 1864^S^

# Hydrozoa

Hydroids [Hyd]^I,S^

*Aglaophenia* sp.

*Antennella* sp.

*Clytia* sp.

*Sertularella* sp.

*Ectopleura* sp.

# Anthozoa

*Actinia equina* (Linnaeus, 1758)^I^

*Aiptasia mutabilis* (Gravenhorst, 1831)^I,S^

*Anemonia viridis* (Forskål, 1775)^I,S^

*Balanophyllia europaea* (Risso, 1826) [Beu]^S^

*Caryophyllia smithi* Stokes & Broderip, 1828^S^

*Cereus pedunculatus* (Pennant, 1777) [Cpe]^I,S^

*Cladocora caespitosa* (Linnaeus, 1758)^S^

*Corynactis* sp.^I^

Unidentified Actinians^I,S^

# Polychaeta

Serpulidae^I,S^

# Polyplacophora^I^

# Bivalvia

*Gastrochaena dubia* (Pennant, 1777) [Gdu]^I,S^

*Lithophaga lithophaga* (Linnaeus, 1758)^I,S^

Unidentified Bivalves^I^

# Gastropoda

*Dendropoma* spp.^I^ *D. anguliferum* (Monterosato, 1884)

*D. petraeum* (Monterosato, 1884)

*Haliotis tuberculata lamellosa* Lamarck, 1822^S^

*Phorcus turbinatus* (Von Born, 1778)^I^

*Patella* spp.^I^

*Serpulorbis arenarius* (Linnaeus, 1767)^I,S^

*Stramonita haemastoma* (Linnaeus, 1767)^I,S^

Vermetidae^I,S^

# Cirripedia

Barnacles^I,S^ *Balanus* spp.

*Chtamalus* spp.

# Bryozoa

*Calpensia* sp.^S^

Erect Briozoans^I,S^

*Bugula* sp.

*Margaretta cereoides* (Ellis & Solander, 1786)

*Crisia* sp.

*Scrupocellaria* *reptans*(Linnaeus, 1767)

Encrusting Brown Bryozoans^I,S^

*Schizobrachiella sanguinea* (Norman, 1868)

*Schizomavella* sp.

# Ascidiacea

*Ascidia mentula* Müller, 1776^S^

*Botryllus* sp.^S^

*Clavelina lepadiformis* Müller, 1776^S^

*Cystodytes dellechiajei* (Della Valle, 1877) [Cde]^S^

Didemnidae^I,S^

*Didemnum lahillei* Hartmeyer, 1909

*D. granulosum* (Drasche, 1883)

*D. maculosum* (Milne-Edwards, 1841)

*Diplosoma listerianum* (Milne-Edwards, 1841)^I,S^

*Halocynthia papillosa* (Linnaeus, 1767)^S^

*Microcosmus* sp.^S^

*Styela* sp.^S^
